# Genetics in an isolated population like Finland: a different basis for genomic medicine?

**DOI:** 10.1007/s12687-017-0318-4

**Published:** 2017-07-20

**Authors:** Helena Kääriäinen, Juha Muilu, Markus Perola, Kati Kristiansson

**Affiliations:** 1 National Institute for Health and Welfare, Helsinki, Finland; 2 Institute for Molecular Medicine, Finland (FIMM), Technology Centre, University of Helsinki, Helsinki, Finland; 3 Institute for Molecular Medicine, Finland (FIMM) and Diabetes and Obesity Research Program, University of Helsinki, Helsinki, Finland; 4 Estonian Genome Center, University of Tartu, Tartu, Estonia

## Abstract

A unique genetic background in an isolated population like that of Finland offers special opportunities for genetic research as well as for applying the genetic developments to the health care. On the other hand, the different genetic background may require local attempts to develop diagnostics and treatment as the selection of diseases and mutations differs from that in the other populations. In this review, we describe the experiences of research and health care in this genetic isolate starting from the identification of specific monogenic diseases enriched in the Finnish population all the way to implementing the knowledge of the unique genetic background to genomic medicine at population level.

## Introduction

Isolated populations may have a unique genetic background as, by chance, the founder population has initially had a certain assortment of gene variants which, again by chance, may have developed towards enrichment of some of the variants and disappearance of some others. As the population size is small, the possible bottleneck events like epidemics, wars, or hunger have a more profound effect on the gene pool when compared to larger populations. The isolation may be cultural as in the case of Ashkenazi Jews or geographical with Faroe Islands as an example. In case of Finland, both geographical isolation due to the very Nordic position of the country and cultural isolation due to religious and language boundaries have caused enrichment of some disease-causing gene variants and losses of others (Norio et al. 1973).

The special genetic constitution of the Finnish population has had a profound effect on the research of genetics of both rare and common diseases as well as on some health care practices. As genomic tools will be more widely used in the healthcare in the future, the unique genetic background of Finns and other isolated populations can be anticipated to offer exceptional possibilities for implementing genomic medicine at the population level.

## The Finnish disease heritage

Traditionally, a group of 36 monogenic diseases which are more frequent in Finland than in any other population have been named the Finnish disease heritage (Norio, 2003b (III)). All these diseases, most of which are autosomal recessive, with their major clinical and molecular findings are presented at www.findis.org. This website focuses on updating what is known about the mutational background of those diseases. However, in addition to enrichment of some monogenic diseases, there are other features related to the Finnish disease heritage as well. Some autosomal recessive diseases which are rather prevalent in other Western and Nordic European countries are much less prevalent or practically lacking in Finland. For instance, the prevalence of cystic fibrosis is only about 1/10 of that in most of the neighboring countries (Kinnunen et al. 2005). In some other diseases, as prevalent in Finland as elsewhere, the mutational background is different and more homologous in the Finnish population. Lynch syndrome with few founder mutations is a good example of this phenomenon (Lynch and de la Chapelle 1999). Similarly, founder effect has influenced the assortment of variants associated with high or low risk for common multifactorial diseases. Genome-wide patterns of common genetic variation also reflect considerable population substructure in Finns (Jakkula et al. 2008). This genetic substructure may explain differences in the regional prevalence of common diseases such as coronary heart disease (Aalto-Setälä et al. 1991; Tyynelä et al. 2010). Finally, the collection of the monogenic Finnish disease heritage cannot be considered complete and closed as new diseases following the same patterns of enriched founder mutations are still identified (Trotta et al. 2016). New diseases can be expected to be found especially among adults as the initial enthusiasm around the phenomenon was mainly among paediatricians.

### Clinical identification of the diseases

**Fig. 1:**
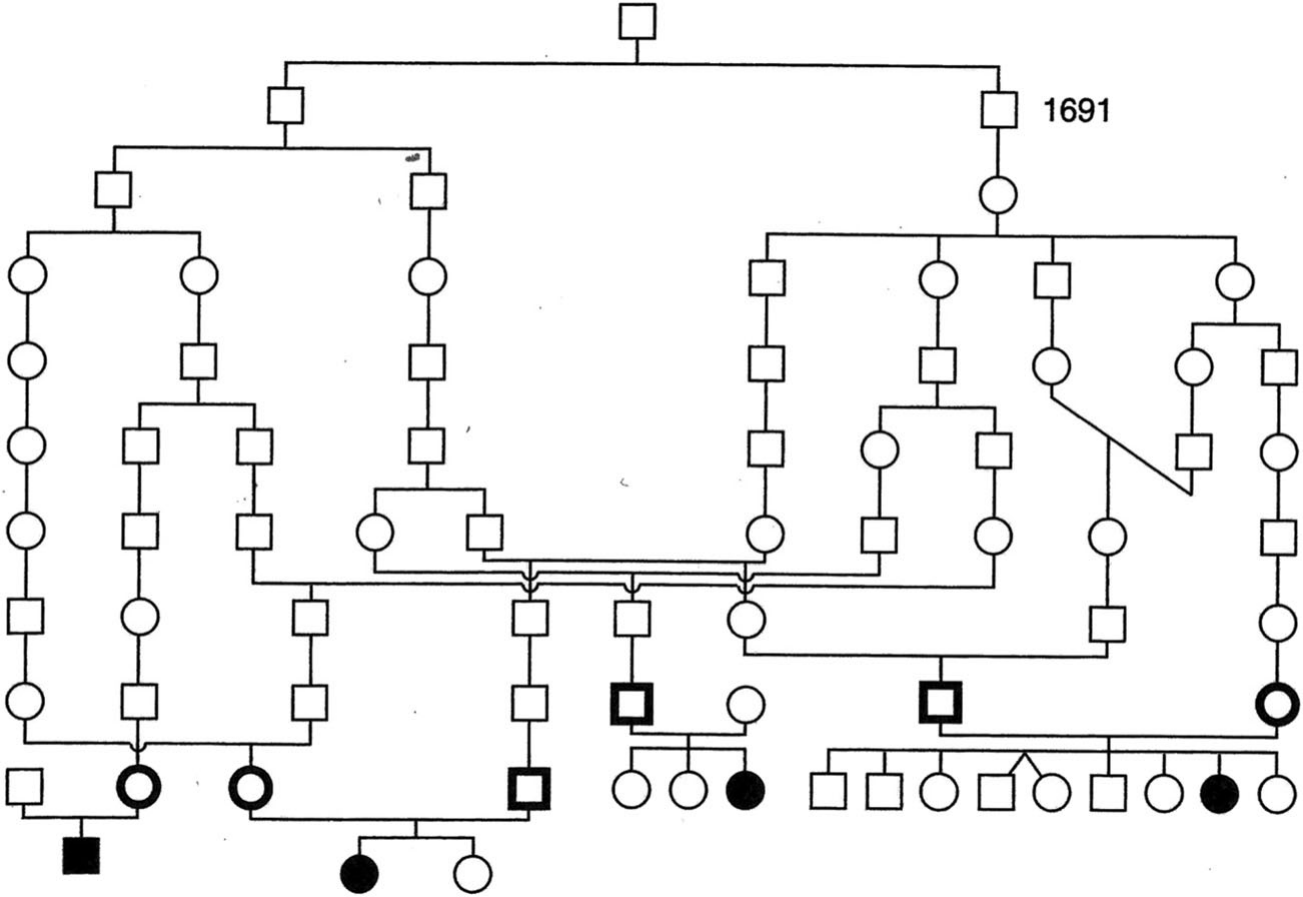
Of the eight parents of four sibships with an autosomal recessive disorder belonging to the Finnish disease heritage, six have been shown using genealogic data from Church Records of Finnish Lutheran Church to descend from an individual who lived in that region some 400 years ago. The figure also shows several more recent connections between the families. Courtesy of Professor Reijo Norio

During 1970–1980 clinical researchers, especially paediatricians and ophthalmologists identified several new disease phenotypes or too many cases of diseases already delineated elsewhere. It started to seem that the assortment of rare diseases, especially autosomal recessive ones, was unique in the Finnish population. During a thorough genealogical study of one of those diseases, congenital nephrosis of Finnish type (MIM # 256300), Norio et al. (1964) realized that instead of several consanguineous marriages like first cousin marriages, as could have been expected as the disease was assumed to be autosomal recessive, there were numerous very remote connections between the parents but also between separate families in early generations even several hundreds of years back (Fig. 1). This led to the understanding that this disease as well as several others was overrepresented in Finnish population and especially in certain regions of Finland because of our population history (Norio et al. 1973). Due to the initially small population, an older founder mutation could, by chance, have been rather prevalent in the founder population of Finland. Later around the sixteenth century, the political decision to inhabit the empty inner land of Finland created a situation where a small sample of individuals became founders of a new regional population carrying their set of mutations with them. As until recently people continued to live in their original rural areas and marry among the surrounding people, this led to a situation where the likelihood of marrying a distant relative was very high, nearly a rule, without the people recognizing that the marriage was actually consanguineous. The situation was accentuated by external forces like wars and periods of hunger creating bottleneck effects which still narrowed the gene pool in each region (Norio 2003a I).

When this was understood, the concept of the Finnish disease heritage started to form. The criterion for a disease to be included in the list was that it should be more prevalent in Finland than elsewhere. However, there never was a strict rule defining how much more prevalent the disease had to be. This kind of rule would have actually been impossible as the epidemiology of rare diseases was and still is poorly known in most countries, including Finland, due to among other things, underdiagnosis. So some diseases maybe were included in the list with too loose criteria and some others were excluded with no good reason. In addition, two of the diseases are inherited in X-linked manner and two are autosomal dominant; for those diseases, the population history does not give similar logical explanation.

**Fig. 2:**
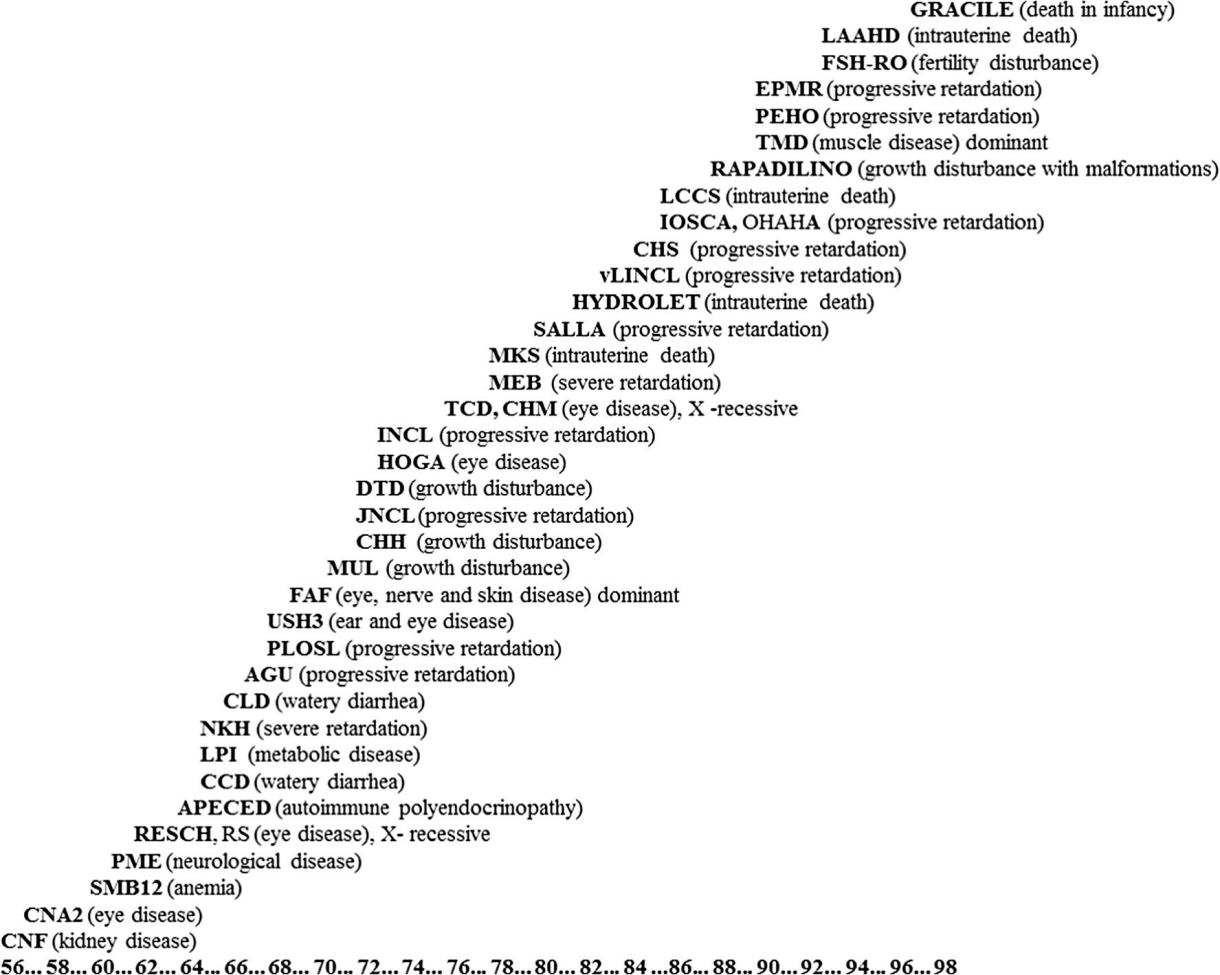
The collection of the diseases of the Finnish disease heritage is often presented as “Perheentupa’s Steps.” This way to illustrate the growing number of these diseases was first used by Professor Jaakko Perheentupa. Each disease is presented at the year when the first Finnish publication of the disease was published. The genes and founder mutations of all these diseases have been detected, nearly always by Finnish researchers. Courtesy of Dr. Teppo Varilo

The diseases on the present official list can be grouped according to the main manifestations. Of them, 11 are progressive central nervous system diseases beginning usually in childhood, 5 are prenatally or neonatally fatal malformation syndromes, 5 are ophthalmological disorder, and 4 manifest with growth deficiency. Many feature multiple symptoms, for instance, Rapadilino patients are small, have several malformations and dysmorphic features and they also have increased risk for some types of malignancies (Siitonen et al. 2009). The diseases have been traditionally presented as “Perheentupa’s steps” as Professor Jaakko Perheentupa suggested this presentation to show how they, one by one during some decenniums, were identified and published as exceptional Finnish cohorts (Fig. 2). In the case of some of the diseases, the initially monotonous phenotype is evolving as today patients are not only detected by their symptoms (leading to targeted genetic testing) but via genomic sequencing of undiagnosed cases.

Phenylketonuria (PKU) is an extreme example of the counter part of the Finnish disease heritage: the diseases that have an exceptionally low prevalence in Finland. When PKU had been characterized, a search was organized among all institutions for mentally retarded in Finland at a time when it was a common practice to care for mentally retarded patients in institutions (Palo 1967). About at the same time, 80,000 newborns were screened for PKU (Visakorpi et al. 1971). Of these attempts to find PKU cases, it was concluded that PKU is much more rare in Finland than elsewhere; the estimated birth prevalence was between 1/100,000 and 1/200,000 while in the neighboring countries, the reported birth prevalence is much higher, about 1/6000 in Estonia (Ounap et al. 1998) and 1/15,800 in Sweden (Ohlsson A et al. 2016). For that reason, Finland offered neonatal screening only for hypothyreosis until very recently.

### Molecular studies: linkage disequilibrium as a tool to locate the genes

In the case of most diseases of the Finnish disease heritage, there were no clues about the pathogenesis which would have helped in trying to identify the genetic defect behind the diseases. Aspartylglukosaminuria (AGU) was an exception as it was known from previous studies that the gene for the enzyme aspartylglucosaminidase (AGA) which is deficient in AGU is located in the long arm of chromosome 4 (Aula et al. 1984). As the disease was shown to be linked to the same region of chromosome 4 (Grön et al. 1990), the next step was to isolate AGA cDNA and identify the Finnish major mutation in the gene (Ikonen et al. 1991). In the remaining autosomal and X-linked recessive diseases, the genes were identified by linkage studies which were usually strongly facilitated by the phenomenon called linkage disequilibrium.

In the case of the relatively young Finnish population, both mutations that were brought to the country with the founder population and mutations that had later occurred within the population can be considered relatively recent in this population meaning that the crossing overs have not had time to separate the mutations from their initial surrounding chromosomal location. The time from the founding of the Finnish population or many of its subpopulations is so short as measured in the number of meiosis, that the DNA stretches flanking the disease genes show much longer areas of linkage disequilibrium than in older populations (Jakkula et al. 2008). This has often helped to locate the disease genes with smaller patient cohorts and thus advanced the final detection of the causative genes and their mutations (de la Chapelle 1993; Peltonen et al. 1999).

As the genes of the Mendelian Finnish diseases were one by one detected, the search for the mutations showed that there was only one major mutation (in 70–100% of alleles) in most of the Finnish monogenic diseases. This obviously facilitated genetic diagnostic already at the time, when sequencing whole genes was beyond normal clinical diagnostics. Details of the mutational background of the diseases can be found at www.findis.org. This website, hosted by Institute for Molecular Medicine Finland, aggregates variation data of these diseases from Leiden Open Source Variation Database (LOVD) (Fokkema et al. 2011), which is one of the most comprehensive databases of mutations and their consequences. The goal is to provide a single entry point to the data maintained in LOVD and provide necessary background information and links to relevant resources. The mutation data is also updated to use HGVS naming standard (http://varnomen.hgvs.org/) and positioned on the latest genomic build (Polvi et al. 2013).

### Towards pathogenesis of the diseases and developing treatment

There have been enthusiastic efforts for elucidating the pathogenetic mechanisms of the diseases of the Finnish disease heritage but, understandably, the main part of the work has happened in Finland. A lot is known about the pathogenesis of already most of these diseases and the research is ongoing. However, the pharmacological industry has not been as eager in searching for treatments and starting clinical trials as in the case of “more common rare diseases.” At present, there are several ways to treat the symptoms of these diseases, including special diets (for instance, fructose intolerance MIM # 229600 and lysinuric protein intolerance MIM # 222700), organ transplantation (congenital nephrosis of Finnish type MIM # 256300) or orthopaedic treatments (diastrophic dysplasia # 222600). Treatment based on specific orphan drugs is still lacking in all of them. In case of especially the progressive encephalopathies, such treatment if started early enough might dramatically change the future of the patients.

On the other hand, pharmaceutical companies show increasing interest in the advantages the genetic profile of an isolated population such as Finland may offer for research of more common diseases. For example, studies of loss-of-function variants and complete gene knockouts, which are enriched in the population, have provided information with potential therapeutic implications for cardiovascular diseases (Lim et al. 2014).

## Carrier screening programs for monogenic diseases: a possibility to support family planning

When a severe disease is diagnosed in a young child, the parents in Finland usually opt for prenatal diagnostics in the next pregnancies. Often, they also ask: Could this have been prevented somehow? The answer in the case of the autosomal recessive diseases of the Finnish disease heritage actually is “Yes” because of the considerable, nearly dominating, role of one or a few founder mutations behind all those diseases would make population carrier screening rather simple to perform.

First suggestions to start carrier screening in Finland and the first trials date back already to some 20 years (Hietala et al. 1995; Kallinen et al. 2001; Pastinen et al. 2001). In the Finnish health care system, the way to perform such screening would be to embed it to the publicly funded public health care where for instance prenatal screening for chromosomal disorders has taken place since late 1980s. This was, however, never started.

One of the reasons not to start screening for the Finnish disease heritage carriers apparently was that none of the Finnish diseases is very common or well known in the Finnish population (Jallinoja and Aro 2000). The carrier frequencies are known to be different depending on the regions but on the level of the whole population, they have been approximated to vary from 1/45 to 1/100–200. A classic example of carrier screening (in an isolated population) is screening for Tay-Sachs carriers among Ashkenazi Jews. As the carrier frequencies is about 1/25 in this population, of the couples, 1/625 would be carrier couples and, without screening, the birth prevalence of new cases would be 1/2500 (Kaback et al. 1993). If compared with AGU in Finland, the carrier frequency has been approximated as 1/65 meaning that the frequency of carrier couples would be only 1/4300 leading to a birth prevalence of AGU of about 1/17,000 or some 3 cases yearly in Finland.

The lower carrier frequency of the Finnish diseases, when compared to Tay-Sachs, Thalassemia in Mediterranean populations or sickle cell disease in some Black populations means that the screening would find less carrier couples, and thus, the cost-effectiveness might not be as favorable as in those classical carrier screening programs (Cao et al. 2002; Autti-Rämö et al. 2005). On the other hand, the mutational background would make the screening easier and also cheaper than other screening programs based on detection of mutations, for instance carrier screening for cystic fibrosis (Weijers-Poppelaars et al. 2005).

As there is no tradition of carrier screening in Finland, a lot of details would need to be discussed before such screening could be started (Henneman et al. 2016). For instance, what is the level of knowledge of this type of diseases in the Finnish population? How much education would be needed to reach the desired informed choice when offering the screening? What would be optimal age/life situation to offer it? The easiest way to reach the couples would be in the publicly funded maternal health care which is used by practically all families. This, however, would reduce the number of reproductive choices as there would already be an ongoing pregnancy: embryo diagnostics or the choice not to have children (with the couple’s own germ cells) would already be missed. On the other hand, if the genomic data accumulating to biobanks or to health care when personalized medicine comes to the clinic would be used for carrier screening, then often the individuals are in such phases of life that carrier screening is not of personal interest.

One argument favoring a nationwide screening program is equality. Already today, there are companies which offer direct to consumers screening for autosomal recessive mutations for future parents. If such offers come from outside Finland, the assortment of genes/mutations/diseases is often not optimal for Finns and buying such tests cannot be advised for Finnish parents-to-come. However, recently, Finnish laboratories have prepared screening panels better suitable for the local needs. The risk is that couples with good education and financial resources will buy these tests while they would be out of reach for another part of the population with less awareness and no possibility to buy (relatively) expensive tests. In Finland, the aim has been and still is that health care services should be available to everybody in as equal a way as possible.

As new and very expensive treatments are being developed to many hereditary disorders, this might affect the possible decision to start national carrier screening. The public health care could either see the cost of treatments too high and promote carrier screening or, on the other hand, consider that possibility to treatment accentuates the difficult ethical questions related to carrier screening, making it a less acceptable option.

There are few examples of populations with as favorable possibilities for carrier screening for (some) of the recessive disorders as the Finnish population. The molecular genetics of the diseases of the Finnish disease heritage are well studied and documented (Polvi et al. 2013) facilitating reliable carrier tests covering founder mutations (Mathijssen et al. 2017). The development of molecular diagnostic techniques in recent years has introduced opportunities for building designated genetic test panels containing custom genetic variation at a relatively low cost. In addition, using genetic information in public health care is now described as a national strategy in Finland (STM 2015). The opinion of the authors of this review is that the time would now be ready to start preparations for offering a nationwide carrier screening program tailored to the Finnish population. Simultaneously, immigrant couples, who would miss a considerable amount of the benefit from screening programs tailored to native Finns, could be offered more generalizable screening programs or genetic tests specific to their country of origin.

## Possibilities for genetic research and personalized risk evaluation for common multifactorial diseases in the Finnish population

Ministry of Social Affairs and Health (STM) in Finland aims to increase the role of genomics in promoting the health and well-being of Finnish population (STM 2015). Finland’s new genome strategy lists activities which ensure reaching this goal. Integrating genomic research closely to healthcare and promoting the use of genetic risk profiling are among those key activities. Pilot projects of personalized risk evaluation for example in coronary heart disease are already carried out in public-private partnerships (https://www.fimm.fi/en/research/ongoing-collaborative-projects/personal-genomics-projects).

Utilization of genetic risk information in predictive medicine requires preceding research to identify genetic loci and eventually genetic variants that associate with the risk of the complex diseases. Typically, study samples need to comprise of tens or even hundreds of thousands of individuals to find more common genetic variants with modest effects on multifactorial diseases. Isolated populations seem to offer substantial advantage for this research; a reduced allelic heterogeneity, and relatively high frequencies of loss-of-function variants and other deleterious mutations facilitate studies of clinically meaningful low-frequency variation (Lim et al. 2014; Zeggini 2014). In some founder populations, it is also reasonable to expect that variants can be discovered with a tenfold lower sample size (Zuk et al. 2014; Chheda et al. 2017). Unique genetic profile of an isolated population may, however, benefit from customized tools such as population-specific genotype imputation panels for its research (Surakka et al. 2010).

In addition to specific genetic background, in Finland, genetic research has gained significant advantage from access to detailed genealogical records and the possibility to link study participants to national health registers which follow the health of Finns longitudinally. These features also promote developing personalized risk evaluation in preventive healthcare. Large Finnish prospective cohorts with genome-wide genotype data are available for testing the clinical significance of polygenic risk scores or single genetic variants at the population level. Reduced phenotypic and environmental heterogeneity are expected to make the risk estimates more readily applicable to the whole population. These risk estimates are on the other hand population-specific: population-wide changes in exogenous factors, such as the shift from boiled to filtered coffee and other dietary changes in Finland (Pietinen 1996), also characterize the overall risk evaluation.

Finally, the large existing and upcoming genotyped sample collections in Finnish biobanks together with the Biobank Law enable recall of subjects by genotype. This introduces valuable opportunities for personalized preventive medicine in Finland.

## Is the profile of incidental findings different in an isolated population?

The wealth of genomic data emerging from research in the era of genome-wide genotyping and sequencing has prompted discussion of the need to report incidental genetic findings in the course of genetic diagnostics or genomic research projects. These findings are separate from the original purpose of the genomic investigation but have potential health or reproductive significance to the person studied. Health implications of these secondary findings range from pharmacogenetic responses to disease-predisposing or disease-causing genetic variants. Most debate today concerns returning information of rare pathogenic variation in genes causing preventable disorders, for instance, cardiac arrhythmias or cancers (Green et al. 2013; Kalia et al. 2017).

Profiles of incidental genetic findings in an isolated population may be different compared to more outbred populations. Mutations causing rare recessive disorders may be much rarer or more frequent in a population isolate. Autosomal dominant diseases do not need isolation to occur but can still be regionally concentrated as is the case with Familial amyloidosis, Finnish type in Finland (Meretoja 1973). In rare diseases of both recessive and dominant inheritance, the disease gene in question often holds a few founder mutations that are responsible for majority of the disease events in the population isolate. In Lynch syndrome, for instance, three founder mutations cover approximately 60% of the syndrome carriers in Finland, meaning that it would be possible to detect the majority of the approximately 5000–10,000 Lynch syndrome carriers by screening only the founder mutations (Nyström-Lahti et al. 1995). The situation may turn out to be similar in other diseases as well: there may exist a few globally recurrent mutations complemented with an assortment of Finnish mutations in our population. (Karami et al. 2013; Puurunen et al. 2013). Searching for carriers of the known few founder mutations and returning information to them in the course of diagnostics or genomic research projects could have a significant impact in preventive healthcare. On the other hand, as our population is becoming more diverse, linking a population isolate to genetic disease may lead to unequal access to testing and personalized medicine (Brandt-Rauf et al. 2006).

## Is the Finnish disease heritage disappearing in the future?

Of the 5.5 million inhabitants of Finland today, nearly 40% lives in cities. Especially, young people move to cities for higher education and/or employment. This means that the parents of today’s and future children less often originate from the same regional isolates in the rural areas. In addition, in the recent years, the immigration has greatly increased so that today one in ten of people aged 25 to 44 and living permanently in Finland are of foreign background (Statistics Finland http://tilastokeskus.fi/index_en.html).

This development means that in coming decenniums, the prevalence of the autosomal recessive diseases belonging to the Finnish disease heritage is likely to diminish even though the founder mutations will remain in the population of Finnish origin. Similarly, the variants associated with common multifactorial diseases will slowly become less homogeneous. However, a considerable part of the population still represents genetically typical Finns and even continues living in the regional isolates. So the health care has to flexibly respect and implement the experience of the Finnish genetic research but be prepared to also take into account the different needs of the genetically distinct parts of the population (Valle 2012). For genetic discoveries for which performing research in our isolated population would be beneficial, the time to proceed is optimal just now: the genetic knowledge and the technologies are ready for this research and the main part of the population still represents the original genetic isolate.

## The role of genetic diversity in implementing genomic medicine

Recognition of Finnish population as a genetic isolate increased interest to genetic studies and was also reflected in national research funding some 20 years ago in Finland. Understanding and utilizing the unique genetic profile of Finns has since led researchers to identify specific diseases of the Finnish disease heritage, to find associations between genetic variants and common diseases in the Finnish population, and finally to provide valuable data on sequence variants in Finns for both researchers and clinicians (Sequencing Initiative Suomi, The 1000 Genomes Project Consortium 2015). When research in Finns and other special populations add valuable information to the etiology of both rare and common diseases, it will not only help the populations concerned but subsequently promote research, medical implementations, and drug development on a more global level.

Including genetic diversity in studies searching for associations between genome and diseases may facilitate but also impede research unless the allelic heterogeneity is considered in the analysis. The genetic profile of a disease may differ between populations which could weaken the power to detect associations and lead to failed attempts in replicating the results in other study samples. On the other hand, as the disease-associated genes and variation in them may be different in different populations, utilizing the diverse genetic profiles valuably contributes to the overall quest of piecing together information on the complex biological pathways leading to diseases.

The new possibilities of genomic medicine, where genetic information from research and clinical sequencing directly promotes the health of individuals, require detailed knowledge of the genetic profile of the population in question. To reduce inequality in genetic screening and treatment opportunities between populations and ethnicities, back ground data of genetic diversity and its meaning needs to be collected and utilized.
